# Environmental RNAi pathways in the two-spotted spider mite

**DOI:** 10.1186/s12864-020-07322-2

**Published:** 2021-01-09

**Authors:** Mosharrof Mondal, Jacob Peter, Obrie Scarbrough, Alex Flynt

**Affiliations:** 1 Cellular and Molecular Biology, University of Southern Mississippi, Hattiesburg, MS USA; 2 School of Plant Sciences, University of Arizona, Tucson, AZ USA

## Abstract

**Background:**

RNA interference (RNAi) regulates gene expression in most multicellular organisms through binding of small RNA effectors to target transcripts. Exploiting this process is a popular strategy for genetic manipulation and has applications that includes arthropod pest control. RNAi technologies are dependent on delivery method with the most convenient likely being feeding, which is effective in some animals while others are insensitive. The two-spotted spider mite, *Tetranychus urticae,* is prime candidate for developing RNAi approaches due to frequent occurrence of conventional pesticide resistance. Using a sequencing-based approach, the fate of ingested RNAs was explored to identify features and conditions that affect small RNA biogenesis from external sources to better inform RNAi design.

**Results:**

Biochemical and sequencing approaches in conjunction with extensive computational assessment were used to evaluate metabolism of ingested RNAs in *T. urticae*. This chelicerae arthropod shows only modest response to oral RNAi and has biogenesis pathways distinct from model organisms. Processing of synthetic and plant host RNAs ingested during feeding were evaluated to identify active substrates for spider mite RNAi pathways. Through cataloging characteristics of biochemically purified RNA from these sources, *trans-*acting small RNAs could be distinguished from degradation fragments and their origins documented.

**Conclusions:**

Using a strategy that delineates small RNA processing, we found many transcripts have the potential to enter spider mite RNAi pathways, however, *trans*-acting RNAs appear very unstable and rare. This suggests potential RNAi pathway substrates from ingested materials are mostly degraded and infrequently converted into regulators of gene expression. Spider mites infest a variety of plants, and it would be maladaptive to generate diverse gene regulators from dietary RNAs. This study provides a framework for assessing RNAi technology in organisms where genetic and biochemical tools are absent and benefit rationale design of RNAi triggers for *T.urticae*.

## Background

RNAi is a mode of post transcriptional gene silencing where small 18–30 nucleotide (nt) RNAs associated with Argonaute (Ago) proteins bind to complementary transcripts [1]. In animals, small RNAs are separated into three major classes; microRNAs (miRNAs), small-interfering RNAs (siRNAs), and Piwi-interacting RNAs (piRNAs) [2]. While a role for miRNAs in gene-regulatory networks is highly conserved the function of siRNAs and piRNAs varies by clade. When RNAi is used to inhibit gene expression in invertebrates a common strategy is delivery of long dsRNA for processing into siRNA class small RNAs by the RNase III enzyme Dicer [3]. Unlike most animals, arthropods have multiple Dicers, which at least in *D. melanogaster* segregates miRNA biogenesis from siRNAs [4]. A potential benefit of distinct miRNA/siRNA pathways could be to allow rapid evolution of siRNA behavior for antiviral or genome defense, something that is incongruent with conservation of miRNA function [5]. Organisms distant from *Drosophila* show distinct small RNA biology, which is most apparent in chelicerates such as the two-spotted spider mite, *Tetranychus urticae*, the subject of this study [6–8]. Hundreds of different crops and ornamental plants are infested by spider mites. These mites can rapidly develop resistance to conventional pesticides, making this organism a prime candidate for new pest control technologies such as RNAi [9].

A consequence of the rapidly evolving nature of siRNAs is inconsistency in RNAi efficacy across arthropods. For example, long dsRNA feeding is effective in beetles, but not in moths and butterflies [10]. Exploiting sensitivity to dsRNA in beetles has already yielded commercial products to combat the western corn root worm [11]. However, similar products are not yet available for other pests. This suggests that differences in RNAi response is a major complication that limits application of gene silencing technology. In such an application, animals will need to ingest molecules like long dsRNA, which adds further barriers such as destruction of molecules in gut lumen prior to entry into cells [12]. Moreover, preventing incidental entrance of exogenous RNA into RNAi pathways is likely a desirable trait, especially for polyphagous arthropods which ingest myriad RNA structures and sequences. Thus, characterization of exogenous RNA processing is beneficial for identifying candidate RNAs for technology development. In this study, methods appropriate for non-model organisms that lack genetic tools are used for dissecting small RNA biogenesis. The framework of the approach here would be valuable for examining RNA processing in other non-model organisms.

The small RNA effectors of RNAi are particularly amenable to high-throughput sequencing approaches due to their short length. Fragmentation is not part of sequencing library creation, so RNA ends created by RNases like Dicer are preserved. Also due to sequential ligation of adapters strand is preserved. By analyzing read sizes, nucleotide content, and overlaps between complementary, alternate-strand mapping reads; class and biogenesis patterns can be elucidated. In arthropods, Dicer products (siRNAs and miRNAs) are typically 19–23 nt long. The RNase III domain of Dicer causes staggered cleavage of dsRNAs that leaves 2 nt 3′ overhangs between strands of siRNA duplexes. In contrast, piRNAs are represented by longer 25-30 nt reads. Biogenesis of piRNAs involves cleavage by piwi proteins guided by the activity of pre-existing piRNAs. Ago and Piwi proteins may exhibit slicer activity which cuts the base of target transcript 10 bases from the 5′ end of the bound small RNA. After piwi cleavage, the fragmented transcript is converted into a new piRNA directly or by alternative nucleases. Each of these biogenesis modes can be determined through sequencing data analysis independent of genetic tools.

To evaluate efficiency of feeding-based RNAi in the two-spotted spider mite, *Tetranychus urticae*, an approach was used that combines a purification method alongside detailed analysis of small RNA sequencing libraries. Several studies have tested *T. urticae* for sensitivity to dsRNA, finding saturating exposure like soaking in dsRNA solution is required for robust phenotypes [13, 14]. This suggests further investigation of small RNA populations in *T.urticae* could lead to insights that could lead to superior RNAi techniques. Furthermore, mites as with other chelicerae arthropods, harbor RNA-dependent RNA polymerases (Rdrps), a factor in nematodes and plants that potentiates RNAi through amplifying dsRNA. However, there is little functional evidence that animal Rdrps, other than those found in nematodes, have a role in RNAi pathways that interact with exogenous RNAs [15–17]. Additionally, spider mites unlike *D. melanogaster* have a central role for siRNAs in genome surveillance [6, 18].

By applying this strategy, we find a variety of exogenous RNAs are processed into small RNAs following ingestion by spider mites. Interestingly, the most active configuration is not the expected long dsRNA. We find various plant produced-RNAs are converted into small RNAs after ingestion. However, very few seem to associate with regulatory complexes, something that is affected by feeding behavior and prior exposure to dsRNA. This suggests a scenario where foreign RNAs appear to transit the spider mite RNAi pathway but are diverted from regulatory complexes, which is modulated by the status of the mite physiology.

## Results

**Fig. 1:**
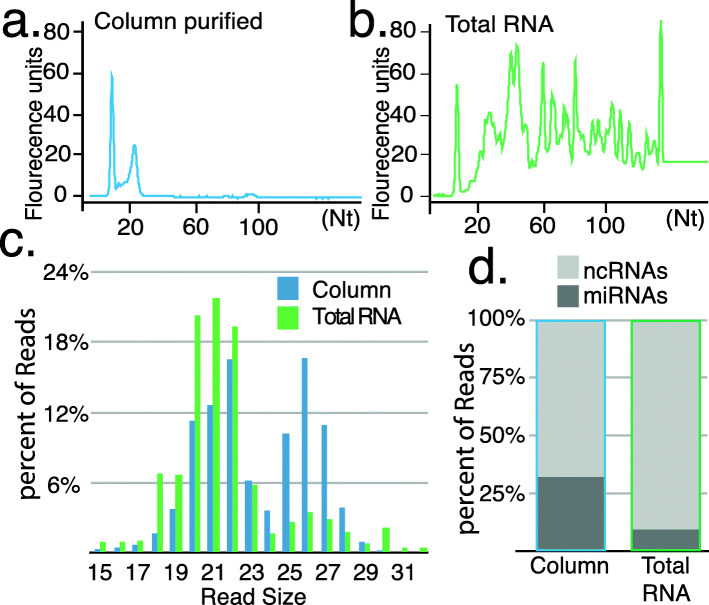
Enrichment of small RNAs with Hi Trap QFF columns. **a.** Bioanalyzer trace of RNA sizes following column purification compared to (**b**) RNAs extracted with TRI-reagent. **c.** Size distribution of reads from sequencing column purified small RNAs or those cloned from total RNA. **d.** Greater recovery of miRbase miRNAs (microRNAs), relative to ncRNA-derived transcripts (tRNAs and rRNAs).

Analysis of environmental RNAs competent to become *trans*-acting is complicated by fragmented transcripts in samples. Immunopurification of Ago complexes followed by isolation and sequencing of associated RNAs can be used to validate small RNA identity [19]. Unfortunately, validated antibodies necessary for this approach are unavailable for all but a handful of organisms. To address this problem, we used Hi Trap QFF chromatography, which can enrich for Ago/Piwi-associated small RNAs (Fig. 1) [20]. Unbound RNAs in animal lysates are retained on the resin while highly basic Ago/Piwi proteins pass through. When used on spider mite extracts, a single clear peak of small RNAs is observed (Fig. 1a). In comparison, total RNA samples show many additional RNAs (Fig. 1b). After library creation and sequencing, size distribution of column purified RNAs shows a lower proportion of reads under 20 nt and an enrichment of piRNA sized (25-28 nt) species (Fig. 1c). Degradation fragments are typically associated with smaller read sizes, thus greater representation of longer sized reads in the column purified libraries demonstrates the effectiveness of isolating functional small RNAs. We also observed three times the representation of *T.urticae* miRbase miRNAs (Fig. 1d) [21]. Using this purification strategy, bona fide *trans-*acting small RNAs processed from exogenous transcripts can distinguished from fragments.

**Fig. 2:**
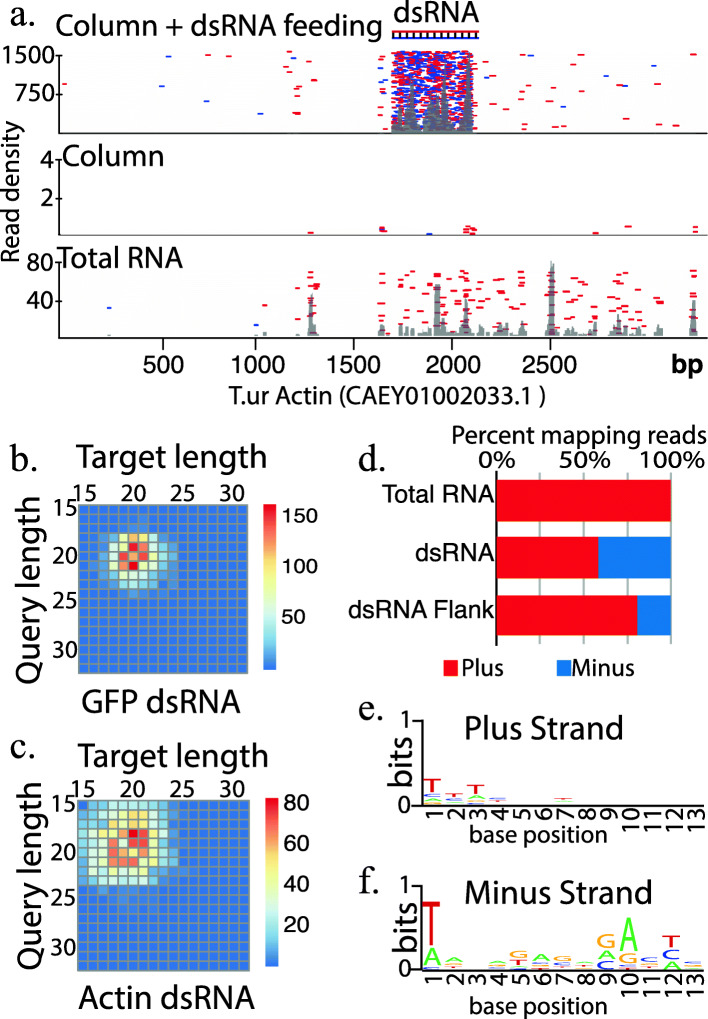
Fate of ingested synthetic long dsRNA. **a** Alignment of reads to the Actin mRNA (CAEY01002033.1) sequenced from column purified RNAs following feeding with dsRNA (top panel), no feeding after column purification (middle panel), and total RNA from unfed mites (bottom panel). Red dashes are positive strand mapping reads, while blue represent negative strand mapping reads. Grey, overlaid density plots represent total read accumulations. **b-c**. Number of Dicer small RNA sequencing read pairs of different sizes derived from dsRNA targeted to GFP (**b**) or Actin (**c**). Pairs are defined by overlapping by 2 nt less than the length of the query strand of the pair. This simulates the 2 nt overhangs found after Dicer processing of dsRNA. Duplexes are biases toward short (18-22 nt) pairs that show asymmetry by one or two bases **d** Percent of plus or minus strand mapping in Total RNA (from bottom panel of 2a), dsRNA targeted region following feeding and column purification (top panel 2a), or flanking region when dsRNA is fed and column purification is used (top panel 2a). **e-f** Seqlogo plot showing sequence bias for reads mapping to dsRNA flanking region (top panel 2a) (**e**) or minus strand (**f**) of Actin

Following previously reported methods, we sought to induce maximum gene silencing using an animal soaking protocol with dsRNA targeted to spider mite Actin and GFP (Fig. 2 & Supplemental Fig. 1). Following exposure to dsRNA, animals were processed into a lysate and small RNAs were isolated with the Hi Trap approach described above. Following sequencing, small RNA reads were aligned to actin and GFP mRNA sequences, which showed significant accumulation of 18–21 nt RNAs at the target region (Fig. 2a & Supplemental Fig. 1). For untreated conditions after Hi Trap enrichment the accumulation was absent with very few alignments. This contrasts with many apparent degradation fragments for total RNA (Fig. 2a). We also found actin and GFP siRNAs were not heritable as none were present in embryos sired by soaked mites (Supplemental Fig. 1b,c).

We then sought to examine Dicer processing of long dsRNA-derived siRNAs by documenting overlaps between complementary pairs of small RNA reads [22]. Specifically, the abundance of small RNA pairs with 2 nt overhangs, characteristic of RNase III cleavage, were summed and plotted in a matrix of pair lengths (Fig. 2b,c). This reveals the sizes of small RNAs found in duplexes that have a signature of Dicer cleavage. In total ~ 45% of Actin and ~ 20% for GFP mapping reads showed signs of Dicer activity consistent with the centrality of this enzyme in the processing of dsRNA, and further validates the purification strategy for examining functional siRNAs. Common pairs were the expected 18–21 nt length and tended to be offset by 1–2 nts (Fig. 2b,c). Actin siRNAs showed more diversity in pair lengths compared to GFP siRNAs, suggesting capture of target cleavage products associated with Ago complexes by Hi Trap purification. We also observed many antisense reads mapping outside the target region that were not present in the total RNA library. This potentially could be the result of Rdrp activity producing antisense transcripts in response to siRNA targeting (Fig. 2d). We then inspected 5′ end sequence biases of actin reads in the dsRNA flanking regions (Fig. 2e,f). While there are no clear sequence motifs in sense mapping reads, antisense have clear 5′ terminal “T” and “A” at the 10 position. This is suggestive of piRNAs, which exhibit these features as a result of piRNA “ping-pong” processing [23]. In spider mites siRNAs act upstream of piRNAs and stimulate their production [6]. These results suggest this may extend to siRNAs derived from exogenous sources. This is consistent with the presence of somatic piRNAs in mites and suggests that piRNA-based processing might participate in eliminating siRNA-targeted transcripts.

Next, we investigated another source of exogenous RNAs–those acquired from plant hosts to assess the universe of RNA species that infiltrate RNAi pathways. Spider mites infest many plants but are readily reared on bean plants (*Phaseolus vulgaris*). In sRNA sequencing libraries created from total RNA extracted from bean-raised animals, ~ 3.13% map perfectly to bean sequences. Of these reads, 85% align exclusively while 15% also map to the mite genome. When Hi Trap purified libraries are aligned to plant sequences there is a 10-fold decrease in mapping rates, and a substantial shift in the proportion of reads that align to both mites and the plant (67%). Hi Trap mite purified RNA was also extracted from animals raised on *Arabidopsis thaliana,* which is not a preferred host of spider mites [24]. We observe reduced plant aligning reads to a rate of 0.09%, consistent with reduced intake of plant materials. 57% of reads from this sample also mapped to the mite genome. Plant mapping RNAs derived from mites have features of endogenous mite small RNAs, such as a peak of read sizes expected for siRNAs (Supplemental Fig. 2). Comparison to public small RNA sequencing libraries from the plants themselves (*P. vulgaris* and *A. thaliana*) showed shifts in the dominant read sizes (Supplemental Fig. 2). Thus, the RNAs in the mite library are unlikely to be mature plant sRNA contaminants.

**Fig. 3:**
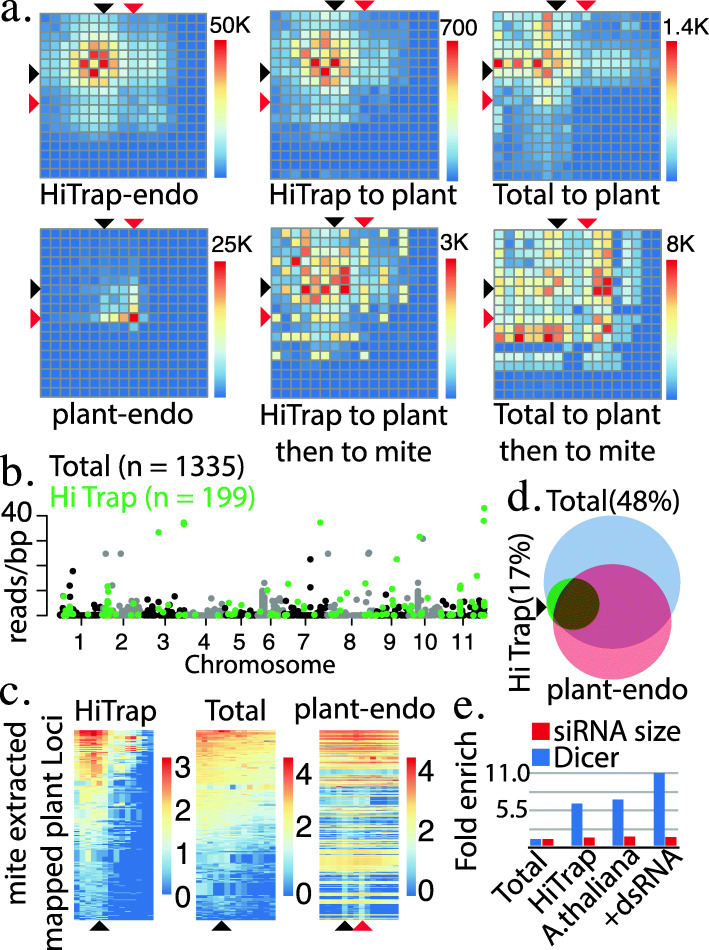
Metabolism of plant RNAs and identities of transcripts that enter small RNA pathways. **a** Number of Dicer overlap read pairs (as defined in Fig. 2b-c) for HiTrap purified mite derived RNAs mapped to the *T. urticae* genome (HiTrap-endo), those mapping to the *P. vulgaris* genome (HiTrap to plant), reads cloned from total RNA mapped to the *P. vulgaris* genome (Total to plant), endogenous plant small RNAs (plant-endo), column purified RNAs first mapped to the *P. vulgaris* genome that are mapped to the *T.urticae* genome (HiTrap to plant then to mite), and total RNA extracted RNAs the *P. vulgaris* genome that are mapped to the *T.urticae* genome (Total to plant then to mite). Black arrow shows 21 nt size RNAs, red arrow 24 nt. Key to right of each graph shows number of reads in each potential pair. Number above represents reads represented by red color, which scales in a linear fashion to dark blue for zero reads. **b** 1335 Loci in *P. vulgaris* (bean) with at least two read depth and greater than 40 nucleotides long identified using total RNAs derived from mites. Loci are plotted by chromosome and number of reads per base pair. Colors of loci alternate between black and grey to distinguish originating chromosome. Green labeled loci are the 199 subset found in HiTrap recovered RNAs from mites. **c** Heatmaps showing size distribution of small RNA reads for *P. vulgaris* loci identified in (**b**). Horizontal of the heatmap is read size ranging from 15 on left and 30 on right. Vertical is individual loci with the highest expressing at top and lowest at bottom. From left is mite-derived HiTrap RNAs (HiTrap), total RNA mite-derived reads (Total), and a bean tissue derived (endo-sRNA) library. Black arrow shows 21 nt size RNAs, red arrow 24 nt. Scales on right are log transformed number of reads. **d** Venn diagram showing overlap between loci plotted in (**b**) and loci expressing endogenous small RNAs. Percentage indicates portion of mite library annotated loci that don’t overlap with endogenous sources of small RNAs. **e** Enrichment of plant mapping reads that exhibit Dicer overhangs in HiTrap purified samples from *P. vulgaris*, *A. thaliana*, and mites fed dsRNA raised on P.vulgaris. Enrichment is calculated as the proportion of reads that exhibit the 2 nt overhang associated with Dicer cleavage. This proportion is normalized to what is observed for total RNA extracted RNAs. The portion of reads between 19 and 23 nt long (siRNA size) is also plotted.

Simultaneously, a substantial portion of the RNAs do appear to be generated from plant transcripts. Dicer overlap pairs from mites derived RNAs that map to plant sequences exhibited a pattern similar to long dsRNA and the mite genome (Fig. 2b,c, Fig. 3a, Supplemental Fig. 3a). The same pattern was not seen with total RNA library mapping to plant (Fig. 3a). Endogenous plant sRNAs were quite different, showing the greatest number of pairs at 24 nt, which is consistent with the 24 nt Dicer products found in plants (Fig. 3a, Supplemental Fig. 3c) [25]. Strikingly, the plant mapping reads from both Hi Trap and total RNA samples do not recapitulate this pattern when aligned to the mite genome (Fig. 3a). Thus, it would seem the plant mapping RNAs derived from mite samples are for a large part produced from plant transcripts despite co-mapping to the *T. urticae* genome. Further, plant mapping reads that do map back to the spider mite genome overlap with longer-sized reads in mites, again potentially tying piRNA production to siRNA targeting in this animal.

To understand the origin of plant derived siRNA found in mite libraries we annotated loci that transmit RNAs to mites based on read alignment density to plant genomes (Fig. 3b, Supplemental Fig. 3d). Using the total RNA dataset, we uncovered 1335 loci and 199 with the Hi Trap sample. Nearly all the Hi Trap loci overlapped with the ones annotated with total RNA, however, this was not tied to relative expression level. Examining the size distribution of reads mapping for each Hi Trap annotated loci showed bias similar to synthetic dsRNAs (Fig. 3 c, Supplemental Fig. 1a, Supplemental Fig. 2c,d). The pattern was not seen for these loci when using total RNA library alignments or a plant endogenous small RNA library (Fig. 3 c). Next, we intersected endogenous plant small RNAs loci with those called from mite derived RNAs libraries (Fig. 3 d). Generally, there was significant overlap with Hi Trap libraries but not total RNA (Fig. 3 d). However, the Hi Trap RNAs are not generated from well-processed plant small RNAs like miRNAs, which were only found in the total RNA dataset. This suggests that while plant RNAs that have features of small RNAs precursors are more likely to enter mite RNAi pathways, it is probably a double-stranded form of the RNA that is taken up. We also observe enrichment of Dicer overlap reads as a portion of mapping reads in Hi Trap samples with an apparent further enrichment for animals exposed to the synthetic dsRNA (Fig. 3 e). Thus, it would appear super abundance of dsRNAs in diet could saturate turnover enzymes and leads to greater stability of the small RNAs derived from dietary sources.

**Fig. 4:**
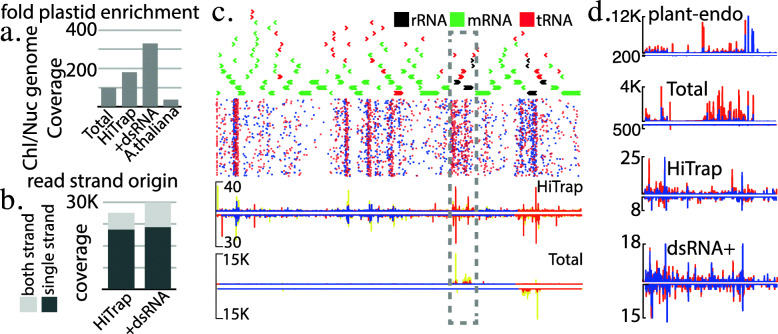
Chloroplast mapping RNAs. **a** Coverage of chloroplast relative to nuclear genome for RNAs recovered from total and HiTrap purified RNAs from mites raised on bean plants, bean plants after dsRNA feeding, and *A.thaliana*. **b** Plastid genome coverage occuring on both strands for a specific region or only on a single strand. **c** Distribution of reads mapping to *P.vulgaris* chloroplast genome. Top trace shows chloroplast genes. Next panel, alignment of individual reads from HiTrap purified samples with red spots corresponding to plus strand and blue negative strand mapping. Bottom two panels show read densities for HiTrap and total RNA samples. Orange represents 19-23 nt reads that map in multiple positions, and blue unique mapping positions for the 19-23 nt size range. Yellow shows densities of all size ranges (15-30 nt) **d** Density plots showing read accumulation in the region in (c) highlighted by the gray dashed box. Library used to generate plot above, mapping separated by strand. Red represents all read ranges (15-30 nt), and blue siRNA size only (19-23 nt). Y-axis is maximum density

To explore the greater accumulation of Dicer products after dsRNA feeding, we focused specifically on alignments of mite derived libraries to plastids as these organelles do not have Dicer activity. Potential dsRNA/shRNA substrates are not processed prior to ingestion [26]. Plastids also lack well defined transcriptional units, leading to potential widespread formation of dsRNA [27]. Consistent with precursors of small RNAs being transmitted we observe over-representation of chloroplast sequences in all mite-derived libraries (Fig. 4a). Greater accumulation was seen in bean fed mites versus *A. thaliana* where less plant material is ingested. Interestingly, we also observe dsRNA feeding correlated with the fold increase in plastid genome coverage. This appears to be in part caused by a shift to dsRNA substrates. The amount of genome coverage for single-stranded mapping is similar between dsRNA fed and non-fed mites while the portion covered by dual strand mapping nearly doubles in the fed animals (Fig. 4b).

Generally, small RNAs map to regions of high gene density and rRNA loci (Fig. 4c) (Supplemental Fig. 4). In total RNA libraries the majority of reads map to a single strand of the rRNA loci. In contrast, the Hi Trap dataset show more consistent dual strand mapping, even at rRNA, albeit at a greatly reduced rate (Fig. 4d). In animals with dsRNA feeding, dual-strand coverage is even more equal with a greater portion of siRNA-sized (19-23 nt) transcripts. This suggests that among plastid RNAs ingested by mites a small, yet clear subset enter RNAi pathway. Prior exposure to dsRNA appears to stabilize the on-pathway RNAs which we propose is the result of saturating processing machinery. Alternately, by targeting Actin the dsRNA treatment could affect gut mobility leading to retention of RNA molecules and potentially infiltration of dsRNA pathways. However, together it would appear that siRNAs generated from exogenous RNAs are very unstable being preferentially eliminated rather than incorporated into regulatory complexes.

**Fig. 5:**
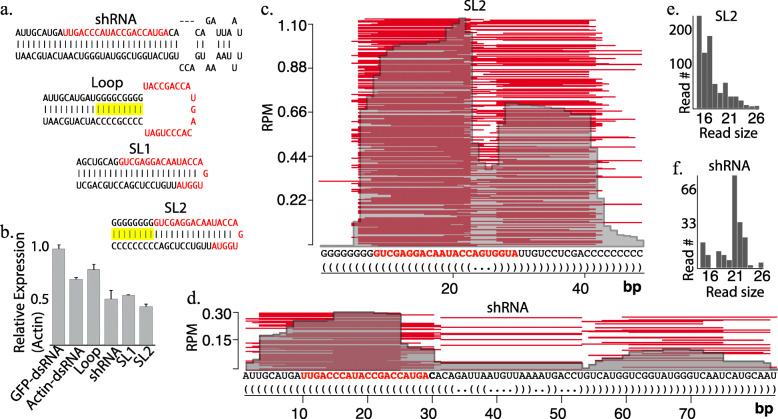
Fate of synthetic short hairpin RNAs fed to spider mites. **a** Actin targeted structured short hairpin RNAs fed to animals via soaking. Designs mimic modes of miRNA biogenesis: Dicer processed (shRNA), Ago processed short loop RNAs (SL1, SL2), and loop derived RNAs (Loop). Red sequence indicates portion targeted to Actin. Yellow highlight show G/C “clamp”. **b** Percent expression of target gene (Actin) following ingestion of long double-stranded RNA (dsRNA) or short RNAs (**a**) determine by qRT-PCR and normalized to rRNA. Error bars are standard deviation. **c-d** Alignment of RNAs sequenced from animals fed SL2 (**c**) or shRNA (**d**) to the synthetic RNA sequences. Red sequence shows portion targeted to Actin. **e-f** Absolute number of reads mapping to short hairpin RNAs SL2 (**e**) and shRNA (**f**)

Based on our finding that structured plant-derived RNAs enter spider mite RNAi pathways we investigated the activity of short hairpin RNAs that mimic distinct, known miRNA-type biogenesis (Fig. 5a). The ability of four different configurations of synthetic hairpin RNAs to trigger silencing of Actin was tested alongside long dsRNA also targeted to Actin by soaking in in vitro synthesized molecules. qPCR was used to quantify target knockdown (Fig. 5b). The short hairpins were designed to transit: canonical miRNA biogenesis (shRNA), Ago processed short loop RNAs that mimic miR-451 with and without a G-C clamp at the hairpin base (SL1 & SL2), and a G-C clamp stabilized RNA with a loop sequence complementary to actin (Loop) [28, 29]. GC clamps were added to the structure bases of the RNAs to increase stability as this high energy fold is a challenging substrate for RNases [30]. After soaking, relative abundance of actin transcripts was assessed showing the reported ~ 40% reduction for long dsRNA [13]. Three of the structured RNAs showed a greater degree of knockdown with a reduction of transcript accumulation reaching nearly 60%, suggesting that the siRNA pathway of *T. urticae* may not be the optimal mode of RNAi to exploit for gene silencing (Fig. 5b).

We also sequenced samples after feeding with the SL2 (Fig. 5c) and shRNA (Fig. 5d) RNAs. SL2 derived small RNAs were more abundant but showed smaller fragments than shRNA, consistent with being an atypical RNAi substrate (Fig. 5e,f). For both RNAs, however, cleavage sites were non-uniform, and for SL2 there was little evidence slicer-mediated precursor processing was occurring as seen with miR-451, but rather standard dicer processing, suggesting that additional engineering could yield an even more superior gene silencing molecule. Nevertheless, many reads were complementary to Actin that were able to trigger knockdown. This together with the appearance of long dsRNA-derived siRNAs shows that a variety of ingested RNAs enter spider mite RNAi pathways, and that the designated siRNA/long dsRNA pathway may not be the most potent option for eliciting knockdown.

## Discussion

Using the strategy described here we were able to provide a comprehensive view of environmental RNA processing in the two-spotted spider mite. Several aspects of small RNA biogenesis emerged that are not found in model organisms to include involvement of ping pong piRNA-like processing of transcripts targeted by dsRNA [8]. Weak Rdrp potential activity was also observed, however, it did not yield a significant population of siRNAs. This was apparent by the lack of transgenerational siRNA inheritance. We were also able to show single-strand structured RNAs are processed by Dicer into small RNAs, which guided the design of several short hairpin RNAs that have greater gene silencing efficacy compared to long dsRNA.

Our analysis has important implications for RNAi strategies for mite plant pests. While we found a wide range of ingested RNAs can access mite RNAi pathways, phenotypes are hard to induce in these animals with RNAi. We propose that this apparent contradiction may indicate that RNAi pathways in mites have another function: metabolism of double-stranded or structured RNAs. Identity of transcripts converted into small RNAs by mites is highly dynamic and tied to feeding state. Moreover, for an animal with hundreds of plant hosts, conversion of ingested RNAs into multi-target gene regulators would be maladaptive. Preferential destruction of small RNAs based on origin is seen in *Drosophila* [31, 32]*.* The mirtron subclass of miRNAs as well as siRNAs derived from latent viruses are poorly recruited to Ago complexes [31, 32]. Navigating such a fate for small RNAs will be critical to effective RNAi technology.

The approach we describe here is species agnostic. There are many species that would be excellent candidates for new pest control technologies where RNAi efficacy is unclear. By evaluating a range of substrates both synthetic and dietary using the strategy we applied to mites, better approaches could be developed. The synthetic hairpin RNAs that showed greater activity in spider mites are likely processed via miRNA biogenesis a much more conserved mechanism than arthropod siRNAs. A similar type of RNAi trigger may be a better choice for animals that have apparent resistance to long dsRNA. The biochemical and computational approach here would confirm this is situation in one of these species.

## Conclusions

This study documents processing of environmental RNAs in the two-spotted spider mite. Insights were possible for pairing a species agnostic biochemical method with sequencing analysis that documented small RNA biogenesis patterns. From this, we were able to observe environmental RNA processing affected by possible Rdrp activity and integration of siRNA targeting with piRNA processing. siRNAs acting upstream of piRNAs had been previously observed to occur with endogenous pathways. Also, by characterizing dietary RNAs from small structured RNAs were found to produce significant knockdown by exploiting miRNA function and not siRNA. These can lead to a better-informed design of RNAi for mite pest species and provides a framework for evaluating environmental RNA processing in other non-model organisms.

## Methods

### Mite handling

Mites were reared on two plants: *Phaseolus vulgaris* or *Arabidopsis thaliana.* Mites were removed from plant leaves by gentle tapping and collected into a microfuge tube. Both juveniles and adults were kept. For column experiments, a volume of 100 μl packed mites was used, which is approximately 500 animals. Mite soaking was done by adding 200 μL of a 160 ng/μL RNA solution to collected animals. Soaking lasted overnight (15–16 h) followed by rinsing in PBS (pH 7.4), and 0.1% Tween 20 solution. Afterwards, mites were collected on a paper towel and allowed to dry before being placed onto a *P. vulgaris* leaf inside of a large petri dish at room temperature. After five days, mites were collected, flash frozen and stored at -80 °C.

### RNA synthesis

For the initial experiment, two dsRNAs were generated from cloned fragments of *T. urticae* actin and of Green Fluorescent Protein (GFP) from *Aequorea victoria* (accession numbers CAEY01002033.1 and FJ172221.1, respectively). Actin long dsRNA and GFP long dsRNA, were both approximately 350 nt long and created using the MEGAscript™ in vitro transcription kit (Ambion). Templates for dsRNA synthesis were PCR products amplified with primers encoding T7 RNA polymerase promoter sequences on their 5′ ends. Following in vitro transcription, lithium chloride precipitation was performed to purify products, and the resulting RNAs were annealed through heating and gradual cooling.

Four synthetic structures were designed based on RNAs shown to enter miRNA pathways. Templates for these structures were created by annealing long, ~ 100 nt, oligonucleotides (supplement). The resultant dsDNAs encoded a T7 promoter at the 5′ end followed by the structured RNA sequence. RNAs were synthesized using the MEGAshortscript™ in vitro transcription kit (Ambion). The synthesis products were purified by phenol chloroform extraction, and ethanol precipitation.

### HiTrap Q FF column enrichment

Column enrichment of small RNA containing complexes used 1 mL HiTrap Q FF columns (GE Lifesciences) as previously described [20]. Briefly, columns were equilibrated following manufacturer instructions. First, 5 mL of start buffer (20 mM HEPES-KOH, pH 7.9) was applied and passed through the column (1 mL/minute). Then 5 mL of elution buffer (20 mM HEPES-KOH, pH 7.9, 1 M NaCl), and 10 mL start buffer (20 mM HEPES-KOH, pH 7.9, 100 mM KOAc) were applied sequentially. Collected animals were washed several times with PBS, pH 7.4, followed by flash freezing and grinding with a mortar and pestle. 1 mL chilled binding buffer (20 mM HEPES-KOH pH 7.9, 100 mM KOAc, 0.2 mM EDTA, 1.5 mL MgCl_2_, 10% glycerol, 0.2% PMSF, 1 mM DTT, 1X Roche EDTA-free protease inhibitor cocktail) was mixed with the pulverized animals. The lysate was then clarified by centrifugation. Cleared lysate was applied to the column and passed at a speed of 1 mL/minute. The column was washed with binding buffer followed by elution buffer (binding buffer with 300 mM KOAc). An equal volume of acid phenol-chloroform was added to the tube, followed by rocking at room temperature. Following phase separation, RNAs were isopropanol precipitated and resuspended in 30 μL of ddH_2_O. Total RNAs extracted from mites used TRI reagent and followed manufacturer protocols.

### RT-qPCR

Total RNA from spider mite samples was used for cDNA synthesis (Thermo Fisher T-7 kit) using random hexamer primers. cDNAs were used in qPCR assays with SYBR Green real-time PCR master mix (Thermo Fisher), following manufacturer protocols. In the qPCR assay, actin transcripts were normalized by assessing levels of 18S ribosomal RNA. Primer sequences for actin and were previously published [13].

### RNA sequencing and analysis

RNA obtained from both column extraction and total RNA extraction were sequenced from an Illumina TrueSeq Small RNA Library Prep Kit library. Sequencing occurred on an Illumina Nextseq500 or Miseq machine using a single-read 50 base pair protocol.

Small RNA TruSeq libraries were initially processed using Fastx toolkit to remove adapter sequences [33]. Libraries were normalized for number of reads by subsampling with the Seqtk program. For experiments looking at dsRNA processing 50 M reads were used, when comparison of plant derived RNAs 14 M were used. Mapping of reads to mite and plant genomes used the Bowtie program with -a -v0 -m200 parameters to find all perfect alignments while also excluding extremely low complexity sequences [34]. Mapping exclusively to actin and GFP sequences used -v0 for only perfect matching RNAs, and chloroplast alignments used -v 0 -a to uncover all perfect alignments.

Alignments were then processed with Samtools and Bedtools to find regions of expression based on read density and merging of juxtaposed features [35, 36]. Bedtools was also used to quantify alignments per feature and determine the intersection of genomic loci with library alignments. All mite and plant sequences as well as their annotations were taken from public databases [18, 37–39]. Size distributions were calculated from alignments converted back to “fastq” format, which were then parsed to determine number of reads corresponding to lengths between 15 and 31 nt long.

A python-based algorithm was used to find overlapping read pairs that represent Dicer cleavage produced small RNA duplexes [22]. Small RNAs of 15–31 nts were used to query target pairs, also 15–31 nts long, that exhibited an overlap of 2 nts less than the size of query RNA. The number of reads for each pair was plotted to show a heatmap matrix. Visualizations were created using the R packages: ggplot2, qqman, Pheatmap, and Sushi packages [40–43]. All mite sequencing data can be accessed for the BioProject ID: PRJNA591169. Public libraries used for plant mapping are: SRR7738374 (*P.vulgaris*), and SRR7947145 (*A.thaliana*).

## Supplementary Information

**Additional file 1.**

## Data Availability

All datasets generated during the current study are available in NCBI BioProject repository: https://www.ncbi.nlm.nih.gov/bioproject/PRJNA591169 Additional datasets used in this study were published: Formey D, Iñiguez LP, Peláez P, et al. Genome-wide identification of the *Phaseolus vulgaris* sRNAome using small RNA and degradome sequencing. *BMC Genomics*. 2015;16 (1):423. Published 2015 Jun 2. doi:10.1186/s12864-015-1639-5 Zhao, X., Li, J., Lian, B. et al. Global identification of *Arabidopsis*lncRNAs reveals the regulation of *MAF4* by a natural antisense RNA. *Nat Commun* **9,** 5056 (2018) doi:10.1038/s41467-018-07500-7

## Abbreviations

miRNAs: microRNAs
siRNAs: small-interfering RNAs
piRNAs: Piwi-interacting RNAs
Rdrps: RNA-dependent RNA polymerases

## References

[1] Höck J, Meister G (2008). The Argonaute protein family. Genome Biol.

[2] Okamura K, Lai EC (2008). Endogenous small interfering RNAs in animals. Nat Rev Mol Cell Biol.

[3] Yu N, Christiaens O, Liu J, Niu J, Cappelle K, Caccia S, Huvenne H, Smagghe G. Delivery of dsRNA for RNAi in insects: an overview and future directions. Insect Sci. 2013;20(1):4–14. 10.1111/j.1744-7917.2012.01534.x. 10.1111/j.1744-7917.2012.01534.x 23955821

[4] Lee YS (2004). Distinct roles for Drosophila Dicer-1 and Dicer-2 in the siRNA/miRNA silencing pathways. Cell.

[5] Gammon DB, Mello CC (2015). RNA interference-mediated antiviral defense in insects. Curr Opin Insect Sci.

[6] Mondal M, Mansfield K, Flynt A (2018). siRNAs and piRNAs collaborate for transposon control in the two-spotted spider mite. RNA.

[7] Mondal M, Klimov P, Flynt AS (2018). Rewired RNAi-mediated genome surveillance in house dust mites. PLoS Genet.

[8] Mondal M, Brown JK, Flynt A (2020). Exploiting somatic piRNAs in *Bemisia tabaci* enables novel gene silencing through RNA feeding. Life Sci Alliance.

[9] Dermauw W, et al. A link between host plant adaptation and pesticide resistance in the polyphagous spider mite <em>Tetranychus urticae</em>. Proceedings Nat Acad Sci. 2013;110:E113–22. 10.1073/pnas.1213214110J. 10.1073/pnas.1213214110 PMC3545796 23248300

[10] Ivashuta S (2015). Environmental RNAi in herbivorous insects. RNA (New York, N.Y.).

[11] Zukoff SN, Zukoff AL. Host Recognition Responses of Western (Family: Chrysomelidae) Corn Rootworm Larvae to RNA Interference and Bt Corn. J Insect Sci. 2017;17. 10.1093/jisesa/iex022. 10.1093/jisesa/iex022 PMC5416774 28931155

[12] Spit J (2017). Knockdown of nuclease activity in the gut enhances RNAi efficiency in the Colorado potato beetle, *Leptinotarsa decemlineata*, but not in the desert locust, Schistocerca gregaria. Insect Biochem Mol Biol.

[13] Suzuki T (2017). RNAi-based reverse genetics in the chelicerate model Tetranychus urticae: a comparative analysis of five methods for gene silencing. PLoS One.

[14] Yoon J-S, Sahoo DK, Maiti IB, Palli SR (2018). Identification of target genes for RNAi-mediated control of the Twospotted spider mite. Sci Rep.

[15] Dalmay, T., Hamilton A, Rudd S, Angell, S., Baulcombe, D. C. an RNA-dependent RNA polymerase gene in Arabidopsis is required for posttranscriptional gene silencing mediated by a transgene but not by a virus. Cell 101, 543–553 (2000). 10.1016/s0092-8674(00)80864-8 10850496

[16] Zhang C, Ruvkun G (2012). New insights into siRNA amplification and RNAi. RNA Biol.

[17] Pinzón N (2019). Functional lability of RNA-dependent RNA polymerases in animals. PLoS Genet.

[18] Grbić M (2011). The genome of Tetranychus urticae reveals herbivorous pest adaptations. Nature.

[19] Hauptmann J (2015). Biochemical isolation of Argonaute protein complexes by ago-APP. Proc Natl Acad Sci U S A.

[20] Lau NC (2006). Characterization of the piRNA complex from rat testes. Science.

[21] Kozomara A, Birgaoanu M, Griffiths-Jones S (2018). miRBase: from microRNA sequences to function. Nucleic Acids Res.

[22] Antoniewski C, Werner A (2014). Animal Endo-SiRNAs: Methods and Protocols.

[23] Brennecke J (2007). Discrete Small RNA-Generating Loci as Master Regulators of Transposon Activity in Drosophila. Cell.

[24] Zhurov V (2014). Reciprocal Responses in the Interaction between Arabidopsis and the Cell-Content-Feeding Chelicerate Herbivore Spider Mite. Plant Physiology.

[25] Zhao J-H (2016). Genome-wide identification of endogenous RNA-directed DNA methylation loci associated with abundant 21-nucleotide siRNAs in Arabidopsis. Sci Rep.

[26] Bally J (2018). Improved insect-proofing: expressing double-stranded RNA in chloroplasts. Pest Manag Sci.

[27] Shi C (2016). Full transcription of the chloroplast genome in photosynthetic eukaryotes. Sci Rep.

[28] Yang JS (2010). Conserved vertebrate mir-451 provides a platform for dicer-independent, Ago2-mediated microRNA biogenesis. Proc Natl Acad Sci U S A.

[29] Winter J (2013). Loop-miRs: active microRNAs generated from single-stranded loop regions. Nucleic Acids Res.

[30] Kowalinski E (2016). Structure of a cytoplasmic 11-subunit RNA exosome complex. Mol Cell.

[31] Flynt A, Liu N, Martin R, Lai EC (2009). Dicing of viral replication intermediates during silencing of latent Drosophila viruses. Proc Natl Acad Sci U S A.

[32] Westholm JO, Ladewig E, Okamura K, Robine N, Lai EC (2012). Common and distinct patterns of terminal modifications to mirtrons and canonical microRNAs. RNA (New York, N.Y.).

[33] Hannon, G. J. FASTX-Toolkit. http://hannonlab.cshl.edu/fastx_toolkit*.* (2010). Accesssed 17 Dec 2020.

[34] Langmead B, Trapnell C, Pop M, Salzberg SL (2009). Ultrafast and memory-efficient alignment of short DNA sequences to the human genome. Genome Biol.

[35] Li H (2009). The sequence alignment/map format and SAMtools. Bioinformatics.

[36] Quinlan AR, Hall IM (2010). BEDTools: a flexible suite of utilities for comparing genomic features. Bioinformatics.

[37] Rhee SY (2003). The Arabidopsis information resource (TAIR): a model organism database providing a centralized, curated gateway to Arabidopsis biology, research materials and community. Nucleic Acids Res.

[38] VanBuren R, Mockler TC. In: Vogel JP, editor. Genetics and Genomics of Brachypodium: Springer International Publishing; 2016. p. 55–70.

[39] Schmutz J (2014). A reference genome for common bean and genome-wide analysis of dual domestications. Nat Genet.

[40] Wickham H. *ggplot2: Elegant Graphics for Data Analysis*: springer, VerlagNew York; 2016.

[41] Turner SD. qqman: an R package for visualizing GWAS results using Q-Q and manhattan plots. *bioRxiv*. 2014:005165. 10.1101/005165.

[42] Kolde R. Pheatmap: pretty heatmaps. 2012;61:915.

[43] Phanstiel DH, Boyle AP, Araya CL, Snyder MP (2014). Sushi. R: flexible, quantitative and integrative genomic visualizations for publication-quality multi-panel figures. Bioinformatics.

